# MANAGEMENT OF ACUTE RESPIRATORY DISTRESS SYNDROME IN A CHILD WITH ADENOVIRUS PNEUMONIA: CASE REPORT AND LITERATURE REVIEW

**DOI:** 10.1590/1984-0462/2020/38/2018280

**Published:** 2020-03-16

**Authors:** Felipe Rezende Caino de Oliveira, Krisna de Medeiros Macias, Patricia Andrea Rolli, José Colleti, Werther Brunow de Carvalho

**Affiliations:** a Hospital Santa Catarina, São Paulo, SP, Brazil.; b Universidade de São Paulo, São Paulo, SP, Brazil.

**Keywords:** High-frequency ventilation, Pneumonia, Respiratory Distress Syndrome, adult, Adenoviruses, human, Ventilação de alta frequência, Pneumonia, Síndrome do Desconforto Respiratório Agudo, Adenovírus humano

## Abstract

**Objective:**

To report the case of a child who developed acute respiratory distress syndrome (ARDS) from a pulmonary infection by adenovirus.

**Case description:**

A female patient aged 2 years and 6 months, weighting 10,295 grams developed fever, productive cough and vomiting, later on progressing to ARDS despite initial therapy in accordance with the institutional protocol for ARDS treatment. The child evolved to refractory hypoxemia and hypercapnia, requiring high parameters of mechanical pulmonary ventilation and use of vasoactive agents. In the treatment escalation, the patient received steroids, inhaled nitric oxide (iNO), was submitted to the prone position, started oscillatory high-frequency ventilation (HFOV) and extracorporeal membrane oxygenation (ECMO) was indicated due to severe refractory hypoxemia. During this time, the patient’s clinical response was favorable to HFOV, improving oxygenation index and hypercapnia, allowing the reduction of vasoactive medications and mechanical ventilation parameters, and then the indication of ECMO was suspended. The patient was discharged after 26 days of hospital stay without respiratory or neurological sequelae.

**Comments:**

Adenovirus infections occur mainly in infants and children under 5 years of age and represent 2 to 5% of respiratory diseases among pediatric patients. Although most children with adenovirus develop a mild upper respiratory tract disease, more severe cases can occur. ARDS is a serious pulmonary inflammatory process with alveolar damage and hypoxemic respiratory failure; Adenovirus pneumonia in children may manifest as severe pulmonary morbidity and respiratory failure that may require prolonged mechanical ventilation. Exclusive pulmonary recruitment and HFOV are advantageous therapeutic options.

## RESUMO

**Objetivo:**

Descrever paciente que evoluiu com síndrome do desconforto respiratório agudo (SDRA) a partir de infecção pulmonar por adenovírus.

**Descrição do caso:**

Paciente de dois anos e seis meses, sexo feminino, peso de 10295 g, que apresentou com quadro de febre, tosse produtiva e vômitos, evoluindo para SDRA. Apesar da terapêutica inicial em conformidade com o protocolo institucional de tratamento da SDRA, a criança evoluiu para hipoxemia e hipercapnia refratárias, necessitando de elevados parâmetros de ventilação pulmonar mecânica e utilização de agentes vasoativos. No escalonamento da terapêutica, a paciente recebeu terapias adjuvantes, foi iniciada ventilação oscilatória de alta frequência (VOAF) e indicada oxigenação por membrana extracorpórea (OMEC) pela hipoxemia grave refratária. Nesse ínterim, a paciente apresentou resposta clínica favorável à VOAF, melhorando do quadro ventilatório e possibilitando a redução das medicações vasoativas e dos parâmetros de ventilação mecânica. A paciente recebeu alta hospitalar após 26 dias de internação, sem sequelas respiratórias ou neurológicas.

**Comentários:**

As infecções por adenovírus ocorrem principalmente em lactentes e crianças com menos de cinco anos de idade e representam de 2 a 5% das doenças respiratórias entre os pacientes pediátricos. Embora a maioria das crianças com infecção por adenovírus desenvolva doença leve do trato respiratório superior, casos mais graves podem ocorrer com comprometimento do trato respiratório inferior. A pneumonia por adenovírus em crianças pode se manifestar com morbidade pulmonar grave e insuficiência respiratória com risco de vida, o que resulta na necessidade de suporte mecânico prolongado. O recrutamento pulmonar exclusivo pela VOAF pode ser uma opção terapêutica útil.

## INTRODUCTION

Pediatric acute respiratory distress syndrome (ARDS) is a severe pulmonary inflammatory process accompanied by alveolar damage and hypoxemic respiratory failure.^1^ Although advances in therapeutic approaches over the past two decades have resulted in significant improvement in outcomes, death from pediatric ARDS can still occur in up to 35% of patients.^2^ Invasive mechanical ventilation (IMV) is an essential component of ARDS support, but several adjunctive approaches are used in these patients’ treatment, including steroids, inhaled nitric oxide (iNO), prone position, high-frequency oscillatory ventilation (HFOV), and extracorporeal membrane oxygenation (ECMO).^3^ However, the pediatrics field lacks evidence-based data for appropriate therapy escalation.

We report the case of a child who developed ARDS from difficult-to-manage adenovirus pulmonary infection, but who were submitted to appropriate adjuvant therapy, resulting in better management and treatment success. This shows that HFOV, despite low response rates reported in studies, favored the outcome in this clinical picture.

## CASE REPORT

A two-year and six-month-old female patient, weighing 10295 g, was admitted to the pediatric emergency room with a five-day history of runny nose, fever, productive cough and vomiting. Tests were requested and clavulanate-associated amoxicillin (50 mg/kg) was introduced due to initial suspected diagnosis of bacterial pneumonia, with a hemoglobin of 11.5 g:dL; hematocrit 34.3%; 14,570 mm^3^ leukocytes (neutrophils: 80.7%, eosinophils: 0%, basophils: 0.2%, lymphocytes: 13.6%, monocytes: 5.5%); 476 mil/mm^3^ platelets; and C-reactive protein 6.96 mg/dL (reference value: above 5 mg/dL, indicative of bacterial infections and systemic inflammatory processes). She presented worsening in breathing pattern and was transferred to the Pediatric Intensive Care Unit (PICU), initially receiving support with a high-flow nasal cannula (HFNC). Antibiotic therapy with cefepime (150 mg/kg/day) that was increased due to clinical worsening, with increased respiratory rate (RR). After 48 hours of admission to the PICU, she was positive for adenovirus (collected in nasopharyngeal secretion). All blood cultures were negative.

**Figure 1.**
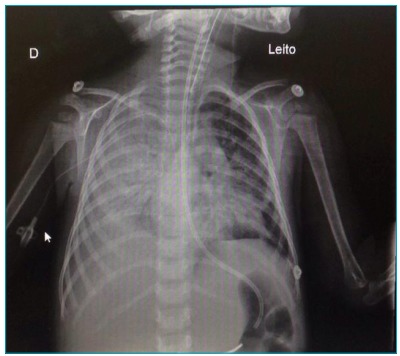
Extensive pneumonia with right hemithorax veiling

She presented radiological worsening with veiling of the right hemithorax (Figure 1). She then received noninvasive MV, without clinical improvement after three hours, and presenting increased RR (65 incursions per minute - ipm) and heart rate (HR) (161 beats per minute - bpm). Orotracheal intubation was chosen by the medical team and conventional mechanical ventilation (CMV) was started with Servo-i^®^ (Maquet, Rastatt, Germany), in intermittent synchronized mandatory mode (SIMV) with pressure support (PS), and Fraction of inspired oxygen (FiO_2_) of 70%; inspiratory time of 0.62 seconds; pressure control (PC) of 17 mmHg; PS of 15 mmHg; positive end expiratory pressure (PEEP) of 8 mmHg; RR of 30 ipm. She was initiated on analgesia with fentanyl (2 mcg/kg/hour) and sedation with midazolam (0.2 mg/kg/hour). Increased ventilatory parameters were required, with PEEP titration up to 12 and FiO_2_ up to 100%.

The patient maintained hypercapnia (carbon dioxide partial pressure - pCO_2_: 98 mmHg) and hypoxemia (oxygen partial pressure - pO_2_: 61 mmHg), with hemodynamic instability, and dobutamine was initially indicated at 5.0 mcg/kg/min, later on increased to 7.5 mcg/kg/min. Norepinephrine was associated after 12 hours due to a decrease in mean arterial pressure up to 44 mmHg at the initial dose of 0.1 mcg/kg/min, which was titrated according to blood pressure and peripheral perfusion parameters up to 0.2 mcg/kg/min.

Two-dimensional Doppler echocardiography was performed, showing ejection fraction of 75%, pulmonary artery outlet pressure of 45 mmHg, mild right ventricular dilation and mild tricuspid regurgitation. The team opted to institute adjuvant therapies. iNO (20 ppm) was initiated; and dobutamine was replaced with milrinone (0.5 mcg/kg/min) due to increased pulmonary artery outlet pressure. The child was placed in prone position due to refractory ARDS for the initial 12-hour period, according to current recommendations reviewed by Koulouras et al.^4^

On the fifth day of evolution, with no clinical improvement, methylprednisolone was used as an adjuvant measure at a dose of 4 mg/kg/day and maintained for 48 hours. Respiratory acidosis and hypoxemia persisted, with average arterial oxygen saturation (SatO_2_) of 77% and pCO_2_>100 mmHg, radiological worsening, and bilateral interstitial pulmonary veiling. Despite the therapeutic support, there was no clinical or blood gas improvement - pH 6.93; pO_2_ 53 mmHg; pCO_2_ 128 mmHg; sodium bicarbonate (BicNa) 26 mmol/l; base excess (EB) -10.6; and SatO_2_ 67%, with oxygenation index (OI) of 39. It was then decided to institute HFOV (Draegger^®^ VN500, Lubeck, Germany) with 100% FiO_2_, frequency of 8 Hz, amplitude of 34 cmH_2_O, mean airway pressure (MAP) of 24 cmH2O, maintaining iNO at the initial dose of 20 ppm and prone position for an additional 12 hours, plus initiation of cisatracurium curarization at 1.2 mcg/kg/min.

Despite the change in ventilatory strategy, initially there was worsening of blood gas parameters (pH 6.87, pO_2_ 66 mmHg, pCO_2_ 169 mmHg, BicNa 30 mmol/L, EB -8.7, SatO_2_ 84%). ECMO was indicated. The patient was already on norepinephrine of 0.4 mcg/kg/min with IO 53. While providing ECMO, cisatracurium curarization was titrated with the train-of-four, showing good synchronization with the VM device. HFOV parameters were changed to MAP of 35 cmH_2_O, frequency of 5 Hz and amplitude of 35 cmH_2_O, maintaining FiO_2_ at 100%.

**Figure 2:**
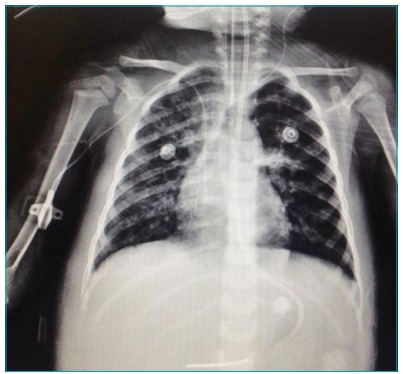
Patient under high-frequency oscillatory ventilation with improvement of acute respiratory distress syndrome, showing reduction on right vein of the right hemithorax in bilateral image.

The patient had progressive improvement and, after eight hours of HFOV, was hemodynamically stable using 0.2 mcg/kg/min adrenaline and 0.4 mcg/kg/min norepinephrine and improved gas exchange (pH 7.23; pCO_2_ 59 mmHg; pO_2_ 85 mmHg; BicNa 24 mmol/L; EB 3.4 and SatO_2_ 96%). After five days, gradual reduction in HFOV began. The patient showed radiological improvement (Figure 2). She was moved after three days to CMV (SIMV + PS) and three days later was successfully extubated.

**Figure 3:**
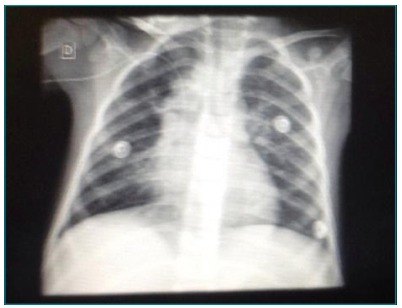
Control X-ray two days after discharge from the Pediatric Intensive Care Unit in room air, with nodular images and regression of lesion previously observed

The child was discharged for home care after 26 days of hospitalization, with adequate saturation in ambient air without respiratory distress. Chest X-ray performed two days after discharge from the PICU showed improvement, with only a few nodular infiltrates in the upper field (Figure 3). The recommendation was Pediatric follow-up for pneumonia because of the severity of the condition. There was no apparent neurological impairment.

## DISCUSSION

Although representing a small proportion of patients admitted to PICU, pediatric ARDS remains a major clinical challenge in intensive care.^1^ Unlike adults, there is a lack of evidence regarding the effectiveness of therapies available for the pediatric age group. Thus, the decision to escalate therapeutic support is extremely difficult and often based on the experience of the multiprofessional team and availability of the hospital’s therapeutic arsenal.^5^

In the case reported, a two-year and six-month-old female patient developed severe adenovirus ARDS. Upon initial evaluation, it is difficult to distinguish adenovirus infection from bacterial infections, which could perhaps explain why antibiotics were prescribed to more than 90% of patients during the study hospitalizations by Shen et al.,^6^ as well as in the case reported. After initial supportive measures with conventional ventilation and no clinical improvement, the patient was placed in prone position. A systematic review of eight randomized studies analyzing prone position in adults undergoing MV showed a reduction in mortality in patients with moderate to severe and longer lasting ARDS (>12 hours).^7^ However, prone position is not free of risks and is associated with increased tracheal tube obstruction and pressure ulcers.

In Pediatrics, there are not enough studies and a consensus is not routinely recommended in patients with ARDS, although it should be considered an option in cases of severe ARDS, as in the case reported.^8^ It is also noteworthy that, in this patient, iNO and steroids for short duration (48 hours) had no success. According to the recommendations of the Pediatric Acute Lung Injury Consensus Conference (PALICC) group, iNO is not recommended as routine in ARDS.^8^ However, it may be considered in patients with documented pulmonary hypertension or severe right ventricular dysfunction. In addition, iNO may be an option in severe ARDS as a “rescue” or bridge to ECMO. Upon its use, benefit assessment should be performed promptly and serially to minimize toxicity and eliminate continued use with no established effect. In the patient reported here, iNO and steroids did not bring the desired therapeutic effect and were discontinued after 48 hours. The decision was then to start HFOV, since the previous measures had showed no clinical improvement.

Despite the lack of consensus in the medical literature,^9^ ^,^ ^10^ ^,^ ^11^ HFOV was effective as a therapeutic measure for this patient, who had already been indicated for ECMO, according to clinical and laboratory criteria. Studies conducted with adults have not shown superiority of HFOV over conventional mechanical pulmonary ventilation (MPV) in ARDS. The Oscillation for ARDS Treated Early (OSCILLATE) study was prematurely discontinued due to increased mortality in the HFOV group.^12^ The OSCilation in ARDS (OSCAR) study reported no difference in mortality between subjects undergoing conventional MPV and HFOV.^13^ In Pediatrics, the Randomized Evaluation of Sedation Titration for Respiratory Failure in High Frequency Oscillatory Ventilation (RESTORE HFOV) study compared, by propensity score analysis, the duration of MPV in pediatric patients with early HFOV (started 24-48 hours after intubation) and those who received conventional MPV or late HFOV.^14^ In this study, early HFOV was associated with longer duration of ventilation but not to mortality compared with those undergoing conventional MPV/late HFOV.

The RESTORE HFOV study seems to have raised more questions than given answers. The recently published European Consensus on Pediatric Mechanical Ventilation^9^ suggests that there is insufficient data to indicate HFOV in pediatric ARDS and that the mode of ventilation should be dictated by clinical experience and theoretical arguments, considering the pathophysiology of the disease. Due to the lack of stronger pediatric consensus, intensive care physicians often decide to use HFOV in pediatric ARDS based on the availability of equipment and the experience of the staff.

Indication of ECMO in severe pediatric ARDS is based on the diagnosis of a previously healthy child without previous non-pulmonary organ dysfunction. The Organization for Extracorporeal Life Support (ELSO) suggests a protocol for indications of ECMO in children that comprises three main clinical conditions:

- Severe respiratory failure (PaO_2_/FiO_2_ ratio <60-80 or OI> 40).
- Lack of response to CMV and other associated therapies (prone position, iNO, HFOV).
- High MV pressures.^15^

In the case reported, the patient presented two of the three necessary conditions after being placed on HFOV. However, after indication of ECMO, there was a time of about eight hours until the availability of the equipment, which was concomitant with the indication of HFOV. Over this period, HFOV parameters were optimized and cisatracurium was started, with substantial clinical improvement: reduction of vasoactive amines, pH (>7.2) and oxygenation improvement, no longer presenting criteria for OMEC. It is noteworthy in this case that, after the optimization of HFOV parameters, there was improvement in clinical and gasometric parameters.

Certainly, the scheduling of therapies presupposes the correct and optimal use of available equipment before opting for the scheduling of therapy. Thus, before indicating the HFOV, it is necessary to make the best possible use of CMV, use PEEP properly, and exhaust the features of the equipment as advanced modes of MV. The same applies to HFOV escalation to ECMO. Equipment needs to be used to the its best before the next step, ECMO - when indicated. This presupposes a properly trained team able to use the equipment resources.

It is also noted that neurological protection is central to the management of critically ill patients and that, despite the severity of the reported case, appropriate clinical management focused on mitigating hypoxemia had a favorable outcome for the patient, who was discharged without apparent neurological or pulmonary sequelae, with discharge for home care in room air without respiratory distress.

It can be concluded that pediatric ARDS remains a challenge for the intensive care physician, mainly due to the lack of scientific evidence related to the therapy being used and high mortality rates. In this case report, the success of treatment was due to the continuous escalation of therapies until the patient achieved clinical improvement with the appropriate use of HFOV in a timely manner, which shows its role in SRDA, although often questioned.
